# CT-based thrombus radiomics nomogram for predicting secondary embolization during mechanical thrombectomy for large vessel occlusion

**DOI:** 10.3389/fneur.2023.1152730

**Published:** 2023-05-12

**Authors:** Shadamu Yusuying, Yao Lu, Shun Zhang, Junjie Wang, Juan Chen, Daming Wang, Jun Lu, Peng Qi

**Affiliations:** ^1^Department of Neurosurgery, Beijing Hospital, National Center of Gerontology, Institute of Geriatric Medicine, Chinese Academy of Medical Sciences and Peking Union Medical College, Beijing, China; ^2^Graduate School of Peking Union Medical College, Beijing, China; ^3^Beijing Hospital, National Center of Gerontology, Beijing Institute of Geriatrics, Beijing, China; ^4^Department of Radiology, Beijing Hospital, National Center of Gerontology, Beijing, China

**Keywords:** stroke, mechanical thrombectomy, thrombus, secondary embolization, radiomics, nomogram, large vessel occlusion

## Abstract

**Background and aims:**

Secondary embolization (SE) during mechanical thrombectomy (MT) for cerebral large vessel occlusion (LVO) could reduce the anterior blood flow and worsen clinical outcomes. The current SE prediction tools have limited accuracy. In this study, we aimed to develop a nomogram to predict SE following MT for LVO based on clinical features and radiomics extracted from computed tomography (CT) images.

**Materials and methods:**

A total of 61 patients with LVO stroke treated by MT at Beijing Hospital were included in this retrospective study, of whom 27 developed SE during the MT procedure. The patients were randomly divided (7:3) into training (*n* = 42) and testing (*n* = 19) cohorts. The thrombus radiomics features were extracted from the pre-interventional thin-slice CT images, and the conventional clinical and radiological indicators associated with SE were recorded. A support vector machine (SVM) learning model with 5-fold cross-verification was used to obtain the radiomics and clinical signatures. For both signatures, a prediction nomogram for SE was constructed. The signatures were then combined using the logistic regression analysis to construct a combined clinical radiomics nomogram.

**Results:**

In the training cohort, the area under the receiver operating characteristic curve (AUC) of the nomograms was 0.963 for the combined model, 0.911 for the radiomics, and 0.891 for the clinical model. Following validation, the AUCs were 0.762 for the combined model, 0.714 for the radiomics model, and 0.637 for the clinical model. The combined clinical and radiomics nomogram had the best prediction accuracy in both the training and test cohort.

**Conclusion:**

This nomogram could be used to optimize the surgical MT procedure for LVO based on the risk of developing SE.

## Introduction

Mechanical thrombectomy (MT) has become a standard treatment for large vessel occlusion (LVO) stroke (1), with a successful recanalization rate ranging from 58.7% to 88% (2, 3). Complete recanalization still cannot be obtained in a small proportion of patients due to the formation of thrombus fragmentation and secondary embolization (SE) during the procedure. Thrombus fragmentation and SE can reduce the anterior blood flow and often require more complex surgical maneuvers to relieve the obstruction, increasing the risk of hemorrhagic transformation (4).

Generally, the stent retriever and contact aspiration remain the first-line thrombectomy strategies in clinical practice (5). However, both techniques can cause thrombus fragmentation (6, 7). These fragments must be curtailed to reduce the risk of emboli and micro-emboli obstructing the downstream cerebral vessels. Several new surgical devices and procedures, such as the balloon guide catheter (8), EmboTrap device (9), Lazarus funnel (10), stent retriever assisted vacuum-locked extraction (SAVE) (11), continuous aspiration before intracranial vascular embolectomy (CAPTIVE) (12), and balloon guide with large-bore distal access catheter with dual aspiration with the stent retriever (BADDASS) (13), can reduce the risk of developing SE and improve the complete recanalization. Nevertheless, their routine use has not been proven to be always necessary or cost-effective. Therefore, there is a need to identify patients at risk of developing SE to optimize the surgical approach for LVO stroke patients.

Clinical and radiological features, such as the use of anticoagulant therapy (14), pre-interventional systemic thrombolysis, and internal carotid artery (ICA) occlusion (15), may play an important role in the development of SE. Furthermore, previous studies have also identified various thrombus features, such as thrombus density and thrombus length, that might be related to the development of SE (16–18). However, these studies did not quantitatively analyze the wide range of features of the whole thrombus that can increase the risk of developing SE.

Radiomics can extract additional features from medical images (19–21) and is increasingly being used to improve the diagnosis and treatment of LVO (22–24). Previous studies used clot-based radiomics features to predict the optimal thrombectomy strategy for successful recanalization (25, 26) and the histological composition of the clot (27). However, to the best of our knowledge, no studies have been conducted investigating the use of CT-based radiomics to predict the risk of SE before MT for patients with LVO stroke. Therefore, in this study, we aimed to develop a prediction nomogram for SE following MT for LVO stroke patients based on clinical and CT-based radiomics features.

## Materials and methods

### Data acquisition

**Figure 1:**
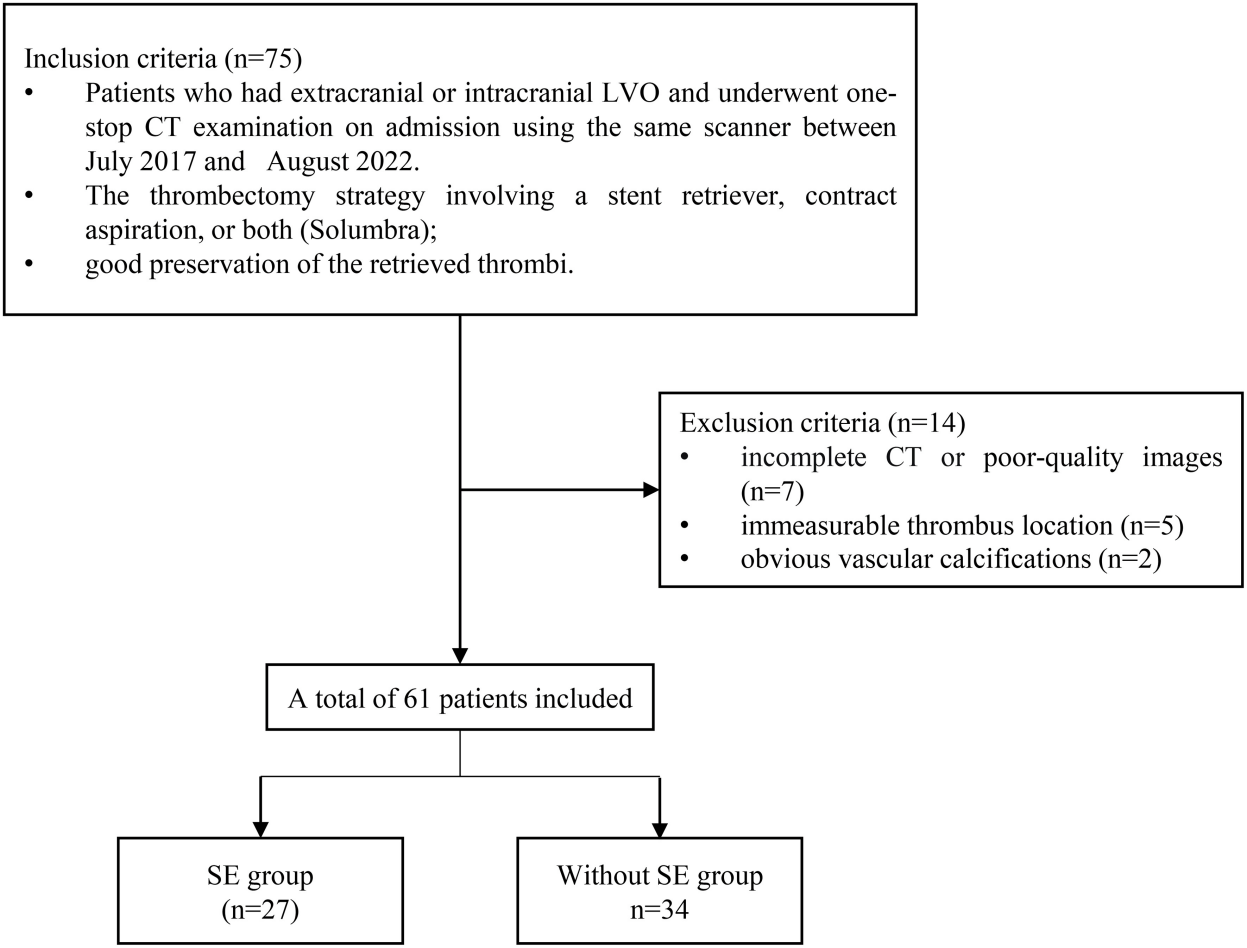
Flow chart for patient enrolment.

**Figure 2:**
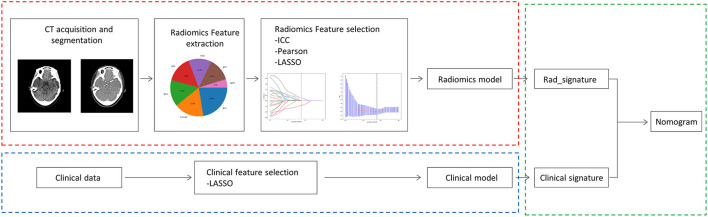
Overall workflow of this study.

The patients with LVO stroke treated by MT at a Beijing Hospital between July 2017 and August 2022 were included in this retrospective study. The patients were included in the study if they had an extracranial or intracranial LVO involving either the anterior or posterior circulation; they underwent a one-stop CT examination that contains computed tomography angiogram (CTA) and computed tomography perfusion (CTP) on admission using the same scanner; they were treated with a thrombectomy strategy involving a stent retriever, contract aspiration, or both (Solumbra); they were with good preservation of the retrieved thrombi. Patients with incomplete CT or poor-quality images that could not be segmented, immeasurable thrombus location, and obvious vascular calcifications were excluded (Figure 1). Subsequently, the patients were randomly divided into training and testing cohorts, in a proportion of 7:3, and the overall distribution of the patient was maintained consistent (the proportion of SE and without SE is the same). The baseline characteristics of the patients, including age, gender, history of stroke, hypertension, hyperlipidemia, diabetes mellitus, atrial fibrillation, current use of anticoagulants, and smoking history; stroke subtype of Trial of Org 10172 in acute stroke treatment (28); conventional thrombus imaging markers, such as thrombus density and the vessel occlusion site, were extracted from the patient's medical records. In addition, the pre-interventional parameters, such as the administration of intravenous thrombolysis therapy, and interventional parameters, including the use of aspiration, stent retriever, Solumbra, and measurements, were also extracted. Figure 2 shows the detailed process of model building.

### Identification of SE

The images of the multiphase CTA acquired on admission and the digital cerebral angiography acquired during MT were reviewed. SE was identified according to the criteria published in our previous report (29). According to these criteria, patients are diagnosed with SE if they have a patent intracranial artery on the admission CTA, or pre-interventional angiography occluded during or after the MT procedure with visible embolisms in either the distal part of the primary occluded vessel or in a completely new location.

### CT data acquisition

Whole brain dynamic volume CTA and CTP were obtained from the Aquilion ONE, Canon Medical Systems CT scanner with 320 × 0.5 mm detector rows and 160 mm coverage. Iopamidol (370 mg*/*ml iodine, Bracco, Italy) or iopromide (370 mg*/*ml iodine, Bayer, Germany) were injected using a high-pressure syringe via the elbow vein*s* with a dose of 0.6 ml/kg, followed by a 30-ml bolus injection of saline. The CTP parameters were 80 kV, 100 mA, and 0.5-mm thickness reconstructed slices. The images were reconstructed using a hybrid*-*iterative reconstruction algorithm and 19 volume data packets, resulting in a total of 6,080 images for each patient.

### Thrombus density measurements

The thrombus density was measured by positioning a region of interest (ROI) at ~2 mm behind the occlusion site, covering approximately half to two-thirds of the vascular area, and another ROI along with its corresponding position of the contralateral artery as explained in our previously reported method (30). The mean Hounsfield Unit (HU) value was recorded on the reconstructed NCCT images for the thrombus and contralateral artery. The relative HU (rHU) (thrombus density/contralateral artery density) and ΔHU (thrombus density-contralateral artery density) were calculated.

### ROI segmentation

**Figure 3:**
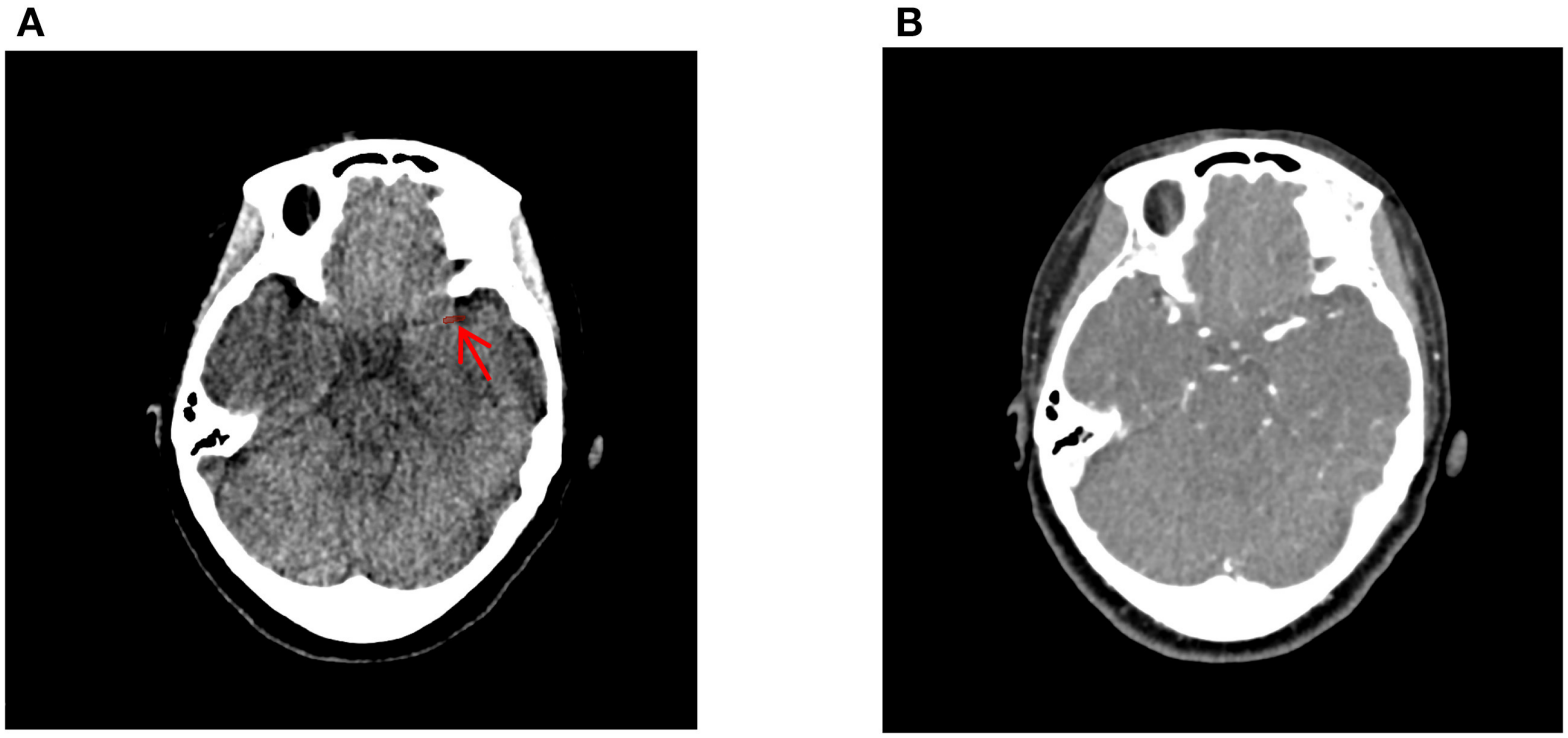
Delineation of the thrombus using the 3D-slicer software. The thrombus on the non-contrast computed tomography **(A)** was manually segmented using the computed tomography angiography image **(B)** as guidance. The red arrow indicates the location of the thrombus ROI.

All NCCT and CTA images of 0.5 mm thickness were loaded into the 3D slicer software version 4.13.0 (https://www.slicer.org/; 3D Slicer image computing platform | 3D Slicer) and registered with ELASTIX [https://elastix.lumc.nl/; elastix (lumc.nl)]. Using the CTA images for guidance, two neuroradiologists (YL and JC) manually segmented the thrombus on each NCCT image (Figure 3). Intra*-*observer and inter*-*observer variability on the segmentation of ROI are presented as intra*-* and inter-class correlation coefficients (ICCs). The detailed process is as follows. In total, 30 cases were randomly selected, then YL and JC independently segmented ROIs during the same period to assess inter-observer agreement. After 1 month, YL repeated manually segmented ROIs again based on the randomly selected 30 cases to assess intra-observer agreement.

### Radiomics features extraction

Radiomics features were automatically extracted from the segmented thrombus using Python's Pyradiomics package (31) (https://pypi.org/project/pyradiomics/). From the NCCT images, 107 radiomics features were extracted. The extracted features were classified into first-order statistics, shape-based, gray-level co-occurrence matrix (GLCM), gray-level size zone matrix (GLSZM), gray-level run length matrix (GLRLM), neighboring gray tone difference matrix (NGTDM), and gray-level dependence matrix (GLDM). These radiomics features are described in detail on the PyRadiomics documentation site (http://pyradiomics.readthedocs.io). All of the above-mentioned features were standardized using the z-score.

### Development of the radiomics signature

The feature dimensions were reduced to minimize radiomics bias, and the course of dimensionality was as follows. The features with a good inter- and intraobserver agreement defined as having an ICC above 0.75 were selected. The Pearson correlation coefficient was then calculated to identify the redundant features. Features with the largest mean absolute correlation or features that had a correlation coefficient of 0.9 or greater were removed. Finally, the least absolute shrinkage and selection operator (LASSO) regression model was used to identify the most useful features based on the training set. Depending on the regulation weight λ, LASSO shrinks all regression coefficients toward zero and sets the coefficients of many irrelevant features exactly to zero. The optimal λ was determined by calculating the minimum cross-validation error following a 10-fold cross-validation. An analysis of retained features with non-zero coefficients was performed to fit the regression models and construct the radiomics signatures. The radiomics score (Rad-score) was then calculated for each patient *via* a linear combination of the selected features weighted by their respective LASSO coefficients. For selecting the optimal radiomics model, different radiomics models were developed and tested, respectively, to predict the risk of SE based on the following eight machine learning classification algorithms: logistic regression (LR), support vector machine (SVM), K nearest neighbor (KNN), random forest (RF), extremely randomized trees (Extra-Trees), eXtreme Gradient Boosting (XGBoost), light gradient boosting machine (LightGBM), and multilayer perceptron (MLP). Then, the SVM machine learning model was identified which has the highest average area under the receiver operating characteristic (ROC) curve (AUC) on the testing set (Supplementary Figure 1). Therefore, the final selected features were inputted into the SVM machine learning models to construct the risk model. A 5-fold cross-verification was performed to obtain the final radiomics signature.

### Development of the clinical signature

The process used to develop the radiomics signature was applied to the development of the clinical signature. The collected clinical features were included in a LASSO regression model to select the most valuable features in the training set, and the features with non-zero coefficients were retained. Then, the selected clinical features were inputted into the same SVM machine learning model to construct the risk model. The final clinical signatures were obtained by 5-fold cross-verification.

### Development of the clinical radiomics nomogram

Logistic regression analysis was used to develop the clinical radiomics nomogram. The diagnostic accuracy of the clinical model, the radiomics model, and the clinical radiomics nomogram was assessed by calculating the AUC in both the training and testing cohorts. A decision curve analysis (DCA) was conducted to evaluate the clinical effectiveness of the clinical radiomics nomogram. The DCA involves calculating the net benefit of a threshold probability range across the training and testing cohorts.

### Statistics analysis

Statistical analysis was performed using the IBM statistical package for social sciences software (SPSS) version 26.0. The Kolmogorov–Smirnov and Shapiro–Wilk tests were used to assess the normality of the evaluated variables. The continuous variables were reported as means ± standard deviations, and the categorical variables were reported as frequency counts and percentages. The chi-square or Fisher's exact tests were used to compare the categorical variables, while the Mann–Whitney U-tests or independent *t*-tests were used for the continuous variables. The Python 3.11.1 software (https://www.python.org) was used for feature extraction and screening and to build the models. The package “regression modeling strategies (rms [R])” was used to develop the nomogram. The metrics used to evaluate the performance of the three nomograms were AUC and 95% confidence interval (95%CI), accuracy, sensitivity, and specificity. The Delong test was used to estimate the differences in the AUC values between the three nomograms. For all statistical tests, a *p*-value below 0.05 was considered to be statistically significant.

## Results

### Patient characteristics

A total of 61 patients were eligible for the study. The training cohort consisted of 42 patients, and the testing cohorts consisted of 19 patients. The clinical and radiological features of all patients included in the study are summarized in Table 1. SE occurred in 27 of the 61 patients. Only large-artery atherosclerosis (*p* = 0.029) and stroke of other determined etiology (*p* = 0.045) differed significantly between the training and testing cohorts.

**Table 1:** Summary of the patients' characteristics in the training and test cohorts.

**Variable**	**Training cohort (*n* = 42)**	**Testing cohort (*n* = 19)**	***p*-value**
Gender, *n* (%)			0.539
Male	23 (54.8)	12 (63.2)	
Female	19 (45.2)	7 (36.8)	
Age, y (mean)	74.52 ± 11.351	71.47 ± 14.946	0.435
Stroke history, *n* (%)	9 (21.4)	6 (31.3)	0.595
Hypertension*, n* (%)	27 (64.3)	10(52.6)	0.388
Hyperlipemia*, n* (%)	9 (21.4)	4 (21.1)	1
Diabetes mellitus*, n* (%)	18 (42.9)	6 (31.6)	0.404
Atrial fibrillation*, n* (%)	19 (45.2)	7 (36.8)	0.539
Anticoagulation*, n* (%)	7 (16.7)	5 (26.3)	0.596
Smoking history, *n* (%)	15 (35.7)	6 (31.6)	0.753
Intravenous thrombolysis*, n* (%)	13 (31.0)	3 (15.8)	0.351
Large-artery atherosclerosis*, n* (%)	28 (66.7)	7 (36.8)	0.029
Cardioembolism*, n* (%)	11 (26.2)	7 (36.8)	0.398
Stroke of other determined etiology*, n* (%)	0 (0)	3 (15.8)	0.045
Stroke of undetermined etiology*, n* (%)	3 (7.1)	2 (10.5)	1
*Internal carotid* artery occlusion*, n* (%)	18 (42.9)	8 (42.1)	0.956
Middle cerebral artery occlusion*, n* (%)	17 (40.5)	8 (42.1)	0.905
*Anterior* cerebral artery occlusion*, n* (%)	2 (4.8)	2 (10.5)	0.777
Basilar artery occlusion*, n* (%)	5 (11.9)	1 (5.3)	0.732
Aspiration*, n* (%)	20 (47.6)	13 (68.4)	0.131
Stent Retriever*, n* (%)	12 (28.6)	2 (10.5)	0.221
Solumbra*, n* (%)	10 (23.8)	4 (21.1)	1
rHU (mean)	1.08 ± 1.45	1.17 ± 0.43	0.789
ΔHU (mean)	6.67 ± 26.34	5.97 ± 18.73	0.918

### Screening and construction of the clinical signatures

After applying the LASSO feature screening, seven clinical features were selected, including smoking history, intravenous thrombolysis, internal carotid artery (ICA) occlusion, stent retriever, Solumbra, rHU, and cardioembolism. These features were used to establish the clinical signature.

### Extraction, selection, and construction of the radiomics signatures

**Figure 4:**
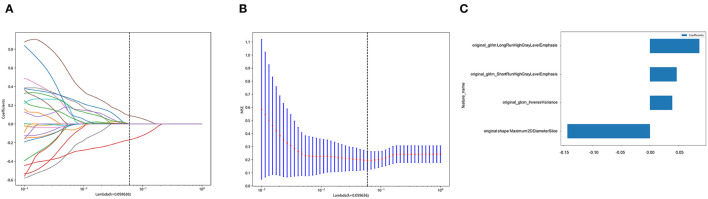
Selection of features based on the least absolute shrinkage and selection operator (LASSO) regression model. **(A)** shows a representative LASSO coefficient distribution map. The vertical dashed line indicates the value chosen after 10 rounds of cross-validation following the coefficient distribution map produced by the λ sequence. **(B)** LASSO model with the adjusted λ parameter following 10 rounds of cross-validation performed to pass the minimum standard. The optimal λ value was 0.059636 and is indicated by the vertical dashed line. **(C)** The selected radiomics features and their corresponding coefficients.

In total, 107 radiomics features were extracted from the reconstructed NCCT images, of which 64 features (ICC >0.75) were found to have satisfactory intra-observer and inter-observer reproducibility. Feature pairs with high correlations were omitted, leaving 27 features per patient for further selection. Finally, four relevant features were identified by LASSO analysis and used to construct the radiomics signature. The best regularization parameter was 0.059636 (Figures 4A, B). Figure 4C shows the selected features and weights. Based on these features, the Rad-score was calculated as follows:

Rad-score = 0.3736422217481793 + 0.038825 × original_glcm_InverseVariance +0.085880 × original_glrlm_LongRunHighGrayLevelEmphasis + 0.046497 × original_glrlm_ShortRunHighGrayLevelEmphasis – 0.143520 × original_shape_Maximum2DDiameterSlice.

### Establishment of the clinical radiomics nomogram

**Figure 5:**
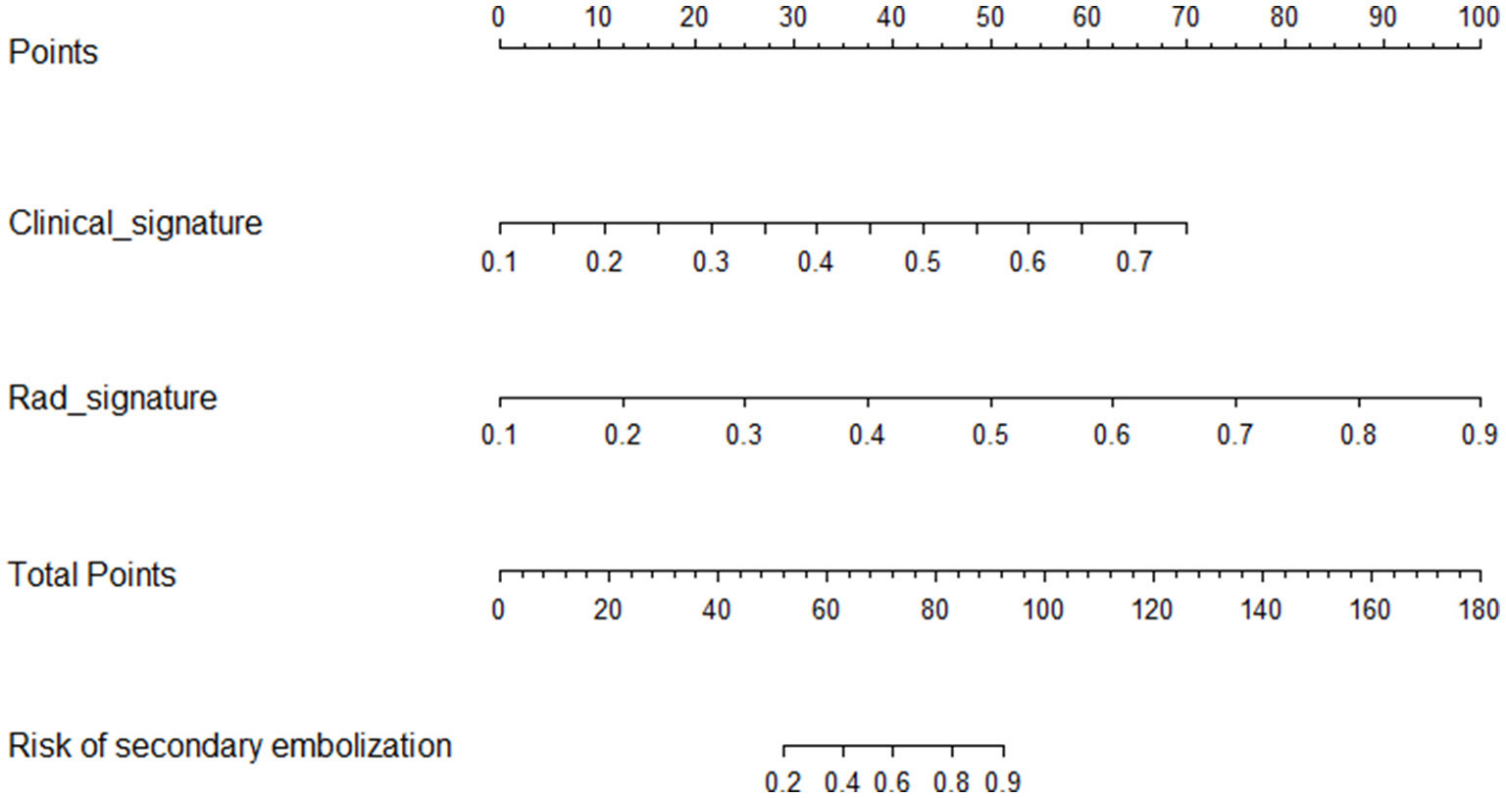
Clinical radiomics nomogram. The radiomics (Rad-signature) and clinical signatures values can be converted into quantitative values according to the corresponding points indicated on the axis. The total risk value was calculated by summing up the individual points. The final sum shown on the total points axis is then used to calculate the overall SE risk.

The combined clinical radiomics nomogram used to calculate the SE risk is illustrated in Figure 5. The risk of developing SE was calculated as follows. A score is first assigned to each influencing factor. Subsequently, all scores are summed up to calculate the total value. A line is then drawn from the total score to the risk axis to calculate the total SE risk. A higher total score is associated with greater SE risk.

### Performance of the clinical, radiomics, and combined nomograms

**Figure 6:**
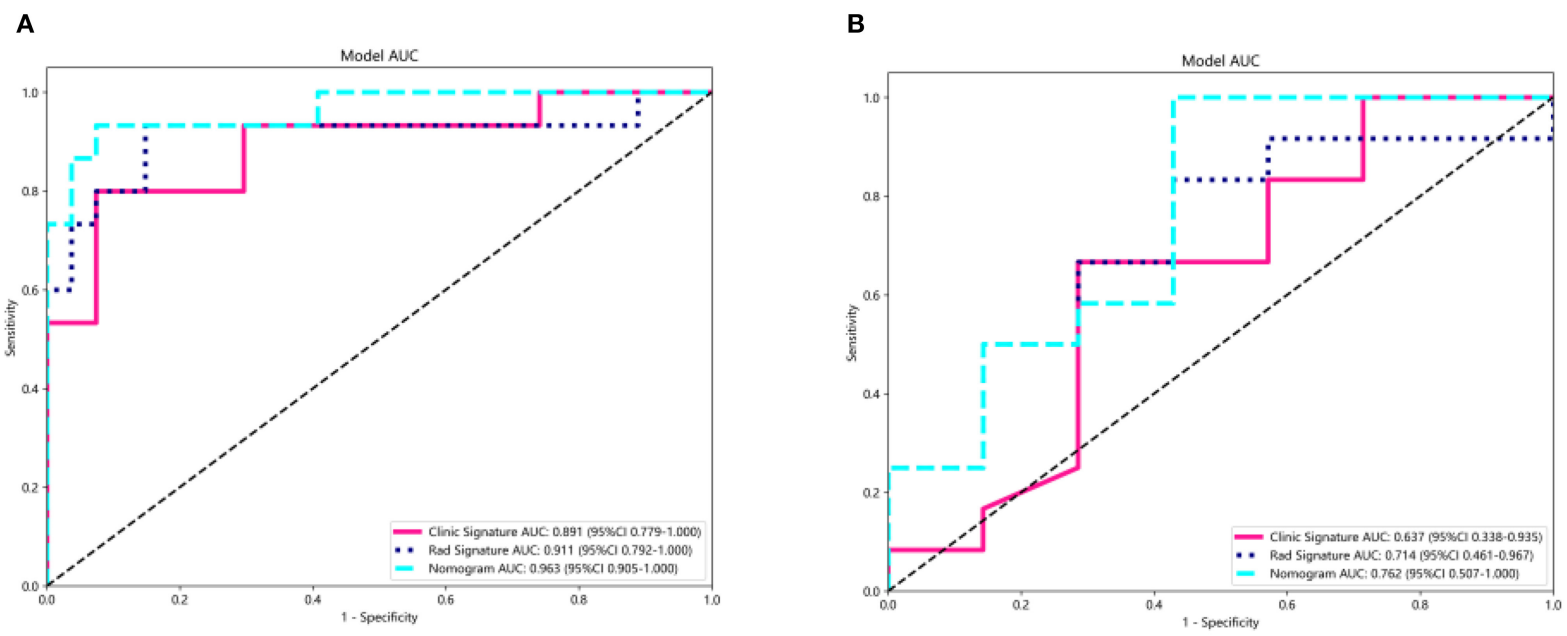
Receiver operating characteristic curves of the radiomics model, clinical model, and the clinical radiomics nomogram for predicting the SE risk for the training **(A)** and test **(B)** cohorts.

A summary of the diagnostic performance of the clinical, radiomics, and combined nomograms is provided in Table 2. In the training cohort, the AUCs of the nomograms were 0.963 for the combined model, 0.911 for the radiomics, and 0.891 for the clinical model (Figure 6A). Following validation, the AUCs were 0.762 for the combined model, 0.637 for the clinical model, and 0.714 for the radiomics model (Figure 6B).

**Table 2:** Performance of the clinical model, radiomics model, and clinical radiomics model in the training and testing cohorts.

**Different models**	**Training cohort (*****n*** = **42)**				**Testing cohort (*****n*** = **19)**			
	**AUC (95%CI)**	**SEN**	**SPE**	**ACC**	**AUC (95%CI)**	**SEN**	**SPE**	**ACC**
Clinical model	0.891(0.779–1.000)	0.800	0.926	0.833	0.637(0.339–0.935)	0.667	0.714	0.421
Radiomics model	0.911(0.792–1.000)	0.933	0.852	0.857	0.714(0.461–0.967)	0.833	0.571	0.579
Clinical radiomics nomogram	0.963(0.905–1.000)	0.933	0.926	0.810	0.762(0.507–1.000)	1.000	0.571	0.632

AUC, the area under the curve; SEN, sensitivity; SPE, specificity; ACC, accuracy; 95% CI, 95% confidence interval.

The Delong test results of the training set showed that no significant difference was noted between the AUC values of the nomogram and the clinical model (*p*-value = 0.191), the nomogram, and the radiomics model (*p*-value = 0.132). In the testing set, there were either no significant difference was noted between the AUC values of the nomogram and the clinical model (*p*-value = 0.266) or the nomogram and the radiomics model (*p*-value = 0.422).

**Figure 7:**
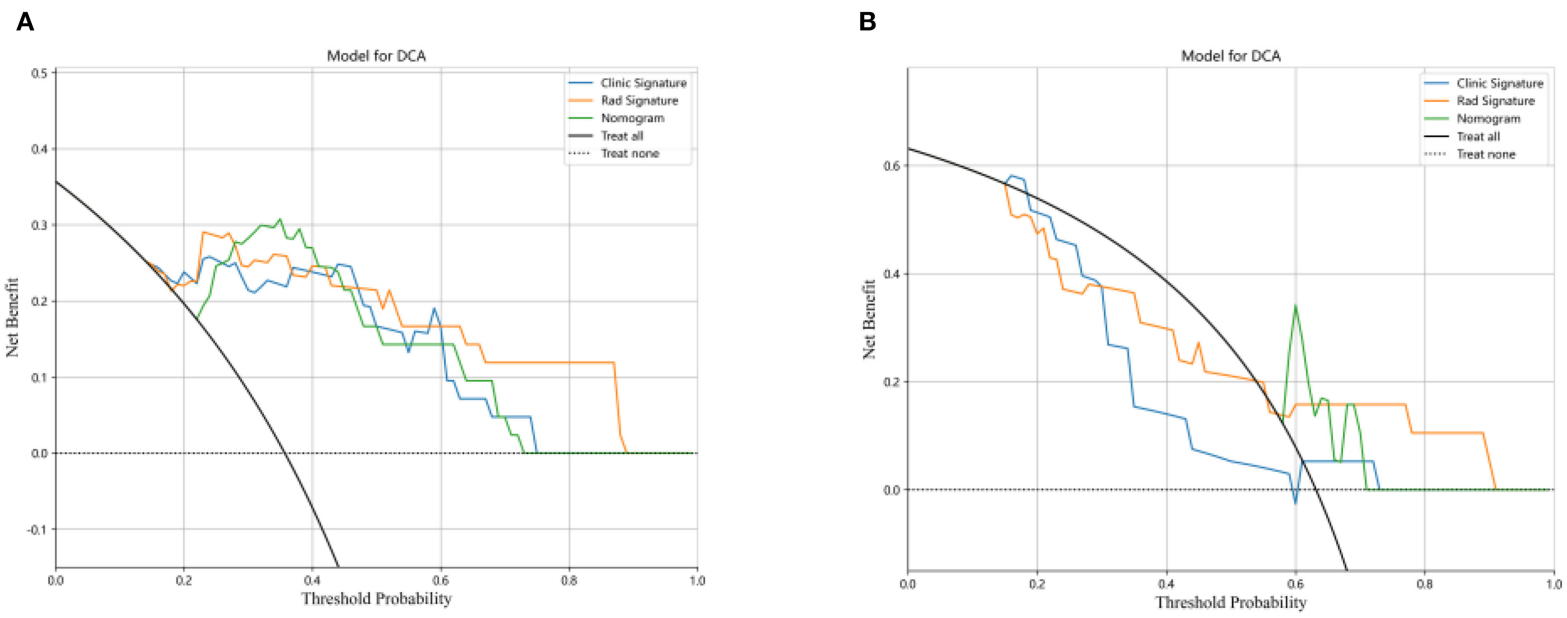
Decision curve analysis for the three models for the training **(A)** and test cohorts **(B)**. The y-axis represents the net benefit, and the x-axis represents the threshold probability.

The DCA for the three models for the training and testing cohorts are illustrated in Figures 7A, B. Compared with the clinical and radiomics nomograms, the DCA revealed that the combined nomogram added a net clinical prediction benefit for most of the threshold probabilities.

## Discussion

Secondary embolization is a common complication of MT. Therefore, there is a need to identify patients at risk of developing SE to optimize the surgical procedure for stroke patients. Previous studies (14, 29) have attempted to develop risk prediction models based on clinical and thrombus features. However, the accuracy of these prediction models varied. To the best of our knowledge, this is the first study using both clinical variables and pre-interventional CT radiomics to identify patients at risk of developing SE.

Studies have shown that the risk of SE is affected by both clinical and thrombus features (32). The clinical information provides important information about the common risk factors, such as the thrombus location and surgical procedure for developing SE. Various studies also evaluated the impact of specific thrombus features on developing SE. In the study of Gengfan et al. (30), thrombus density was identified as an independent predictor of SE. However, this feature does not reflect the full heterogeneities of the different thrombi. The advantage of radiomics is that it can capture a wide range of thrombus features, thus better reflecting the heterogeneity of the thrombus than the conventional thrombus density feature. As a result, under the same SVM machine-learning model, our radiomics model achieved a better performance than the clinical model in both training and testing cohorts. However, the best performance was achieved by the combined clinical radiomics nomogram. The net clinical prediction benefit of the combined nomogram was further confirmed by DCA.

Apart from clinical and thrombus features, other studies used histological thrombus analysis to assess the risk of developing SE. Sporn et al. (18) found that SE occurred more frequently in thrombi with a small fraction of red blood cells. However, the results of the histopathology analysis can only be obtained after the MT procedure; therefore, this data cannot be used to optimize the surgical procedure. Radiomics analysis has the advantage of providing a fast, non-invasive method for neuro-interventional surgeons to objectively evaluate the risk of developing SE (33) before the surgical procedure rather than solely relying on their clinical judgment. The high-risk patients could then have their surgical technique optimized, which may eventually reduce the incidence of SE.

We acknowledge that our study has several limitations. The incidence of SE in our study was 43.3% higher than the 35.2% of patients reported by Gengfan et al. (29), possibly as a result of the selection bias introduced during the retrospective data collection process. The sample was small, and all the data were obtained from one institute. Therefore, a larger multicenter study is required to validate the prediction ability of the nomogram. The manual segmentation of the thrombus is prone to inter-observer and intra-observer variation due to which it can be easily affected by the reader's experience. Automatic or semiautomatic methods could improve the accuracy of the thrombus delineation. Moreover, our model was based on standard radiomics features. A deep learning approach could improve the model's prediction ability. Finally, the underlying biologic meaning of the radiomics features is difficult to interpret. Further studies are required to investigate the correlation between the thrombus histological and radiomics features.

## Conclusion

This study proposed a new clinical radiomics model to predict the risk of SE. The radiomics model showed higher accuracy than the clinical model, and the clinical radiomics nomogram outperformed both the radiomics and clinical models. Our proposed clinical radiomics model could be used by neuro-interventional surgeons to predict the risk of SE, thus allowing them to optimize the surgical procedure according to the patient's needs.

## Data availability statement

The raw data supporting the conclusions of this article will be made available by the authors, without undue reservation.

## Ethics statement

The studies involving human participants were reviewed and approved by the Ethics Committee of Beijing Hospital (2019S-174). Written informed consent to participate in this study was provided by the patient/participants or patient/participants' legal guardian/next of kin.

## Author contributions

SY, PQ, JL, and DW participated in the study design. JW, PQ, and JL were the surgeons and were in charge of patient care. SY and SZ participated in clinical data collection. YL and JC participated in imaging evaluation for AIS patients, delineation of the region of interest, and thrombus density measurement in images. SY, SZ, and JW participated in data analysis, data interpretation, and statistical analysis. SY drafted the manuscript. PQ, JL, and DW revised the manuscript. DW was responsible for the overall supervision. All the authors gave final approval for the version to be published.

## Supplementary material

Supplementary Figure 1 Receiver operating characteristic (ROC) curve (AUC) on the testing set of eight machine learning classification algorithms. ROC, receiver operating characteristic; AUC, the area under the curve; LR, logistic regression; SVM, support vector machine; KNN, K nearest neighbor; RF, random forest; extra-trees, extremely randomized trees; XGBoost, eXtreme Gradient Boosting; LightGBM, light gradient boosting machine; MLP, multilayer perceptron.
