# Supplementary figures and images for: CT-based thrombus radiomics nomogram for predicting secondary embolization during mechanical thrombectomy for large vessel occlusion
Source: Front Neurol. 2023 May 12;14:1152730. doi: 10.3389/fneur.2023.1152730 (PMC10213392; doi:10.3389/fneur.2023.1152730)

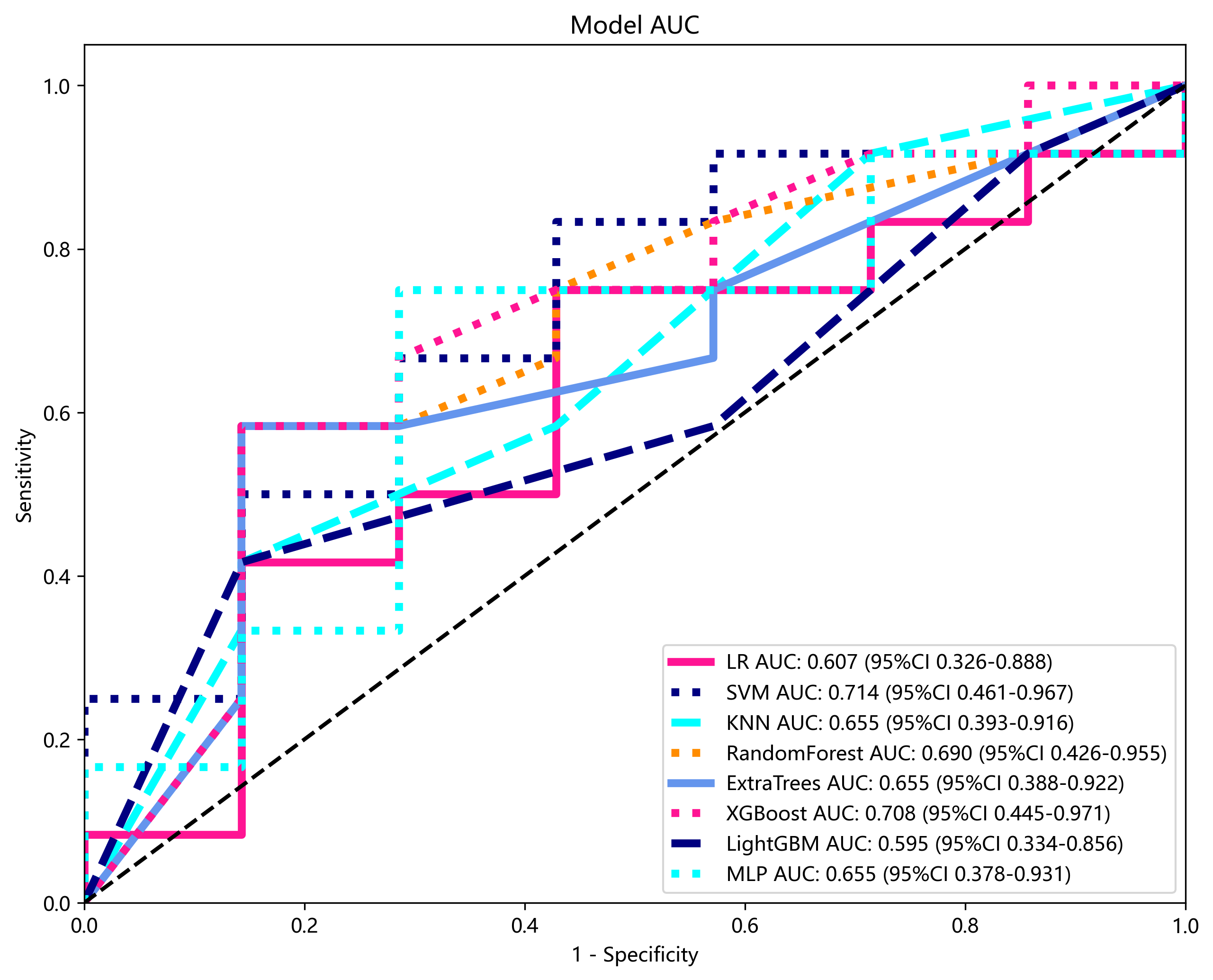

Supplement: Supplementary Figure 1 — Receiver operating characteristic (ROC) curve (AUC) on the testing set of eight machine learning classification algorithms. ROC, receiver operating characteristic; AUC, the area under the curve; LR, logistic regression; SVM, support vector machine; KNN, K nearest neighbor; RF, random forest; extra-trees, extremely randomized trees; XGBoost, eXtreme Gradient Boosting; LightGBM, light gradient boosting machine; MLP, multilayer perceptron. [file Image_1.PNG]
